# Mechanisms of acupuncture in treating depression: a review

**DOI:** 10.1186/s13020-025-01080-7

**Published:** 2025-03-03

**Authors:** Jianfu Ma, Xuan Yin, Kaiyu Cui, Jiqing Wang, Wei Li, Shifen Xu

**Affiliations:** Shanghai Municipal Hospital of Traditional Chinese Medicine, Shanghai University of Traditional Chinese Medicine, 274 Middle Zhijiang Rd, Jing’an District, Shanghai, 200071 China

**Keywords:** Electroacupuncture, Depression, Neuroplasticity, Neuroinflammation, Synaptic remodeling

## Abstract

**Background:**

Acupuncture as a representative treatment method of traditional Chinese medicine, has been found to have a significant effect on mild to moderate depression without obvious side effects, but the mechanism through which it exerts its antidepressant effect is still unclear.

**Methods:**

We searched PubMed, Web of Science, and Embase databases for basic research on acupuncture in the treatment of depression from the database established to June 14, 2024, and finally included 44 studies from 2020 to June 14, 2024, into the table analysis. The main outcomes of this study are the effects of acupuncture on the relevant biological indicators of depression model.

**Results:**

By analyzing the effect of acupuncture on rodent model of depression, the mechanism of acupuncture against depression was explored. In general, several acupuncture methods, mainly based on electroacupuncture (EA), regulate the levels of 5-hydroxytryptamine (5-HT), glutamic acid (Glu) and dopamine (DA), regulate the calcium signaling pathway, increase the expression of synaptic protein, promote mitochondrial repair and reduce oxidative stress, and enhance synaptic plasticity. Inhibition of key inflammatory pathways such as P2X7R/NLRP3 and NF-κB signaling pathways, regulation of hypothalamic–pituitary–adrenal axis (HPA axis) function, and tryptophan metabolism improved depression-like behavior in rodent models.

**Conclusions:**

In summary, acupuncture treatment represented by EA has multiple mechanisms to play a role by regulating neurotransmitter balance, improving neuroplasticity, reducing inflammatory responses, and regulating the neuroendocrine system. However, the differences between acupoint catgut embedding (ACE), manual acupuncture (MA), and EA in the treatment of depression and the operating parameters of EA in the treatment of depression with different causes (such as frequency, intensity, duration, etc.) still need further research to be confirmed.

This review has not been registered with PROSPERO or other protocol registration platforms because protocol registration was not a mandatory requirement for this study.

## Introduction

Depression is the second major disease contributing to the global burden of disease and is well known to the public, with a significant and persistent low mood as its main clinical feature [1]. Patients with major depressive disorder (MDD) are highly heterogeneous in terms of clinical symptoms, biology, and treatment response, and only about one-third of MDD patients can achieve significant remission after treatment [2]. The current clinical guidelines suggest that non-drug methods, such as bio-social psychological methods, should be fully used in the treatment of depression, and drugs should be used in combination for moderate and severe patients.

Therefore, given the current difficulties in the treatment of depression, research to explore safe and effective non-drug therapies is very necessary. Acupuncture is a representative of non-drug treatment in traditional Chinese medicine. In our previous study, 247 patients with depression and insomnia experienced significant improvement in their depressive symptoms and sleep quality after an 8-week EA intervention. The results continued at a follow-up of 24 weeks [3]. The results of the meta-analysis by Smith et al. also showed that acupuncture may bring minor benefits in reducing the severity of depression at the end of treatment, but the quality of the evidence included in the study is very low, and different acupuncture stimulation methods will lead to great differences [4]. Therefore, high-quality randomized controlled trials and further research mechanisms are still needed to test the effectiveness of acupuncture in the treatment of depression.

Although we have previously reviewed the mechanism of EA in the treatment of depression before 2020 [5], the above problems have not been solved. The previous review included depression comorbidity models such as post-stroke depression, which made the results likely to be affected by other comorbidities, and only included EA as a form of acupuncture, not other acupuncture interventions. In addition, there have been many high-quality basic studies on acupuncture treatment for depression in recent years. Therefore, we reviewed the previous basic studies on the treatment of depression by various acupuncture methods, including EA and manual acupuncture (MA), aiming to systematically analyze the mechanism of acupuncture treatment of depression and provide a basis and ideas for acupuncture treatment of depression.

## Methods

### Search strategy

The most commonly used acupuncture methods for clinical treatment of depression are MA and EA [6], so we choose "acupuncture" and "electroacupuncture" as the search terms. Transcutaneous acupoint electrical stimulation, as a new type of electroacupuncture therapy, was also included as a supplement. Finally, in this study, we take "acupuncture," "electroacupuncture," "transcutaneous acupoint electrical stimulation," and "depression" as key words. Articles published in PubMed, Web of Science, and Embase databases from their inception to June 14, 2024, were searched. There are no restrictions on language, document type or publication status.

### Study selection

We selected articles on cellular and animal model studies of acupuncture intervention in depression, excluding other types of articles such as clinical studies and reviews. In order to exclude the influence of other diseases, depressive comorbidity models similar to post-stroke depression were also excluded.

### Data extraction

The two authors conducted a preliminary screening of the title and abstract of the literature independently. After preliminary screening, the literature was screened a second time by reading the full text and then cross-checking the screening results. If there was any disagreement, it was resolved through joint discussion and negotiation.

The study data were extracted and categorized using a pre-made data extraction table that listed the animal model type (strain, type of stress, and species), the type of intervention (stimulation techniques and acupuncture points), and the outcome measures (biochemical measures, behavioral tests linked to depression, physiological indicators, histological indicator analysis, and behavioral tests, etc.). We picked literatures from 2020 to present as representatives and included them in the table.

## Results

**Fig. 1:**
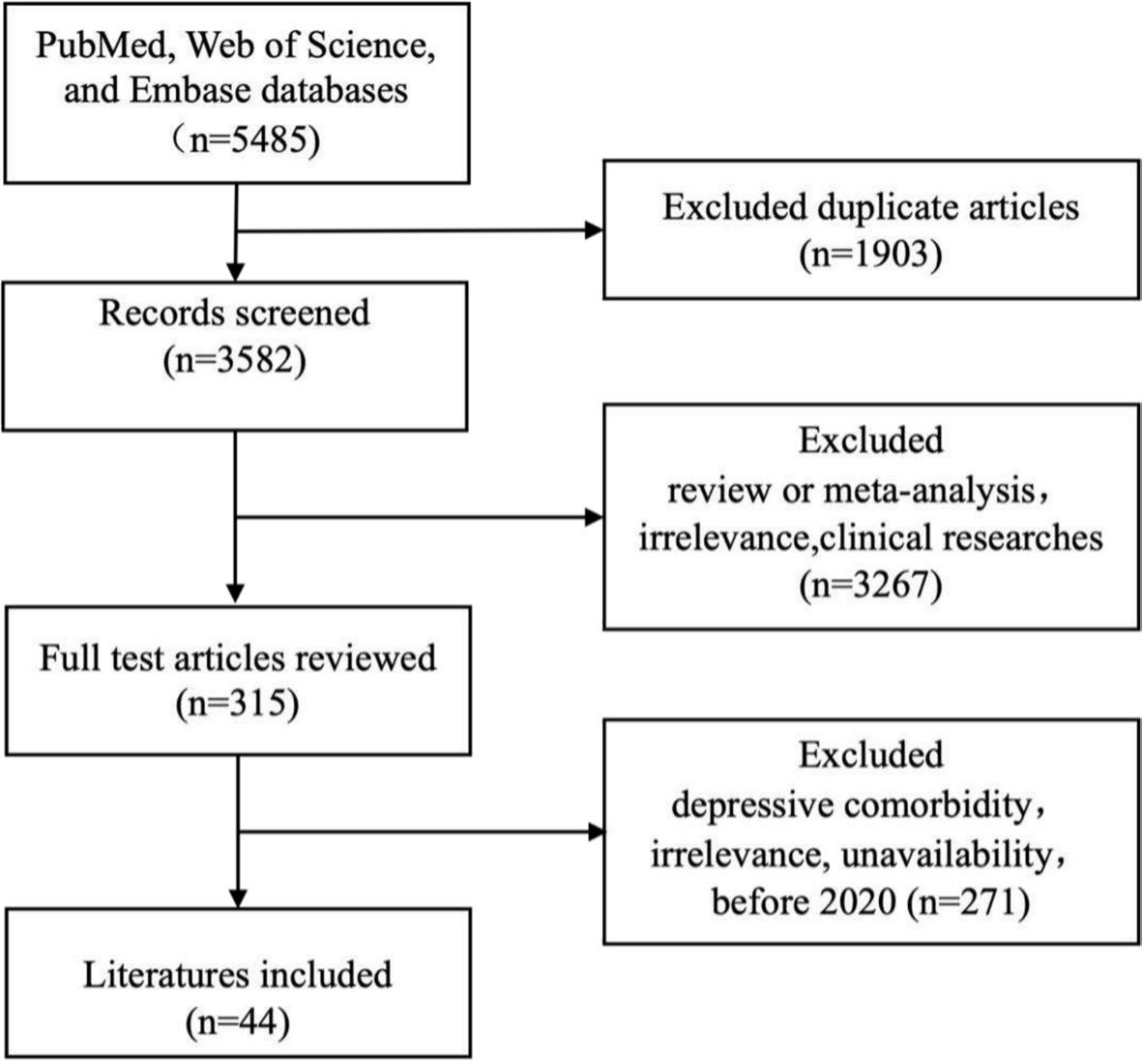
Flow diagram of literature retrieval

A total of 5485 articles were retrieved from the three databases, and a total of 3582 articles were found after eliminating duplicate articles. After we read the titles and abstracts, we excluded reviews, meta-analyses, and other irrelevant articles, totaling 3,267. We then read the remaining 315 articles in full and further excluded a total of 271 articles with depression comorbidity, unavailable full text, before 2020, and irrelevant articles. Finally, 44 published after 2020 were included in the table analysis (Fig. 1).

We analyzed animal models of depression used in 44 studies (Table 1). The chronic unpredictable mild stress (CUMS/CMS) was the most used stress model in these studies, with a total of 29 items. The others are the chronic restraint (CRS) model (5 items), the lipopolysaccharide (LPS)-induced model (2 items), the social defeat stress (SDS/CSDS) model (2 items), the forced swimming (FS) model (2 items), and the social isolation (SI) model (2 items). In the remaining 3 studies, sciatic nerve selective injury (SNI), heterogeneous intermittent stress (HIS), and corticosterone (CORT), respectively, were included in the depression model. The most often utilized behavioral indicators were the sucrose preference test (36) and the open field test (30). The others are the forced swimming test (22), elevated plus maze test (9), tail suspension test (8), novelty-suppressed feeding test (5), social interaction/social interaction rate (2), and thermal pain threshold (1).

**Table 1:** Effects of acupuncture on depressive behavior and its neurobiology in rodents

Model type	Intervention type	Acupoints	Stimulating method	Intervention effect	Biochemical measurements	References
CUMS SD rats	EA	GV20, GV29	2 Hz and 0.6 mA, 30 min, 20 days	SPT: sucrose preference ↑, FST: floating immobility time↓, NSFT: latency time ↓	Aggrecan, brevican, and GAD67 proteins in PrL/IL↑, GLuA1 and PSD95 proteins in PrL/IL↑, PNN cell density in PrL/IL↑	[7]
CRS C57BL/6 J mice	EA	HT7, LR3	2 Hz and 1 mA, 15 min, 7 days	SPT: sucrose preference ↑, TST: immobility time↓	c-Fos in NAc Shell ↑	[8]
SI C57BL/6 mice	EA	GV20, GV29	2/15 Hz and 1 mA, 20 min, one session every other day for 4 weeks	SPT: sucrose preference↑, TST: immobility time↓, OFT: total moved distances↓	P2Y1R in the PFC and hippocampus ↓	[9]
FS-induced mouse	MA	GV20, GV 29	5 mm,15 min,5 days a week for a duration of 2 week	FST: immobility time↓	Preventive: mRNA expression of BDNF, NT-3 and NT-4/5↑; protein expression of BDNF, NT-3 and NT-4/5 ↑,NGF ↓; Therapeutic: mRNA expression of BDNF, NT-3 and NT-4/5 ↑,NGF ↓;protein expression of BDNF, NT-3 and NT-4/5 ↑,NGF ↓	[10]
CSDS C57BL/6 J mice	EA	GV20, GB34	2/100 Hz, 0.1 mA,20 min, 3 weeks	SIT: social interaction ratio↑, FST: struggling time ↑, EPMT: open-arm time ↑, OFT: total moved distances↑	The protein levels of GPR55↑, IL-6, IL-1β, iNOS, and TNFα↓, IL-4 and IL-10 in hippocampus ↑, DCX^+^ and Nestin^+^ cells ↑	[11]
CUMS SD rats	MA, ACE	GV23, GV16	MA: 5 mm,15 min, every other day for four weeks; ACE: 5-mm-long sutures, Once a week, four times in total; 1 h before the CUMS protocol	MA: body weight ↑, SPT: sucrose preference ↑, OFT: total moved distance ↑, NSFT: latency time ↓, FST: immobility time ↓, EPMT: open-arm time ↑; ACE: body weight↑, OFT: total moved distances ↑, NSFT: latency time ↓	MA: 5-HT, FNDC5, IRISIN levels in serum ↑; protein expression of pro-BDNF,BDNF, TrkB,CREB and PKMζ ↑; mRNA expression of BDNF,TrkB and CREB ↑; dendritic spine numbers and length in the LHb ↑	[12]
CUMS C57BL/6 mice	EA	GV20, GV29	2 Hz and 1 mA,30 min, 14 days	body weight↑, OFT: total moved distances ↑, SPT: sucrose preference↑	Relative Lactobacillus abundances ↑, relative abundance of Staphylococcus ↓	[13]
CUMS SD rats	EA	GV20, GV29, BL18	20 min, 2 Hz,6 consecutive days per week for 3 weeks	body weight↑, SPT: sucrose preference ↑, OFT: total moved distance↑, FST: immobility time ↓	Iba1 in the PFC ↓, the expression of P2X7R, NLRP3, and IL-1β related protein in PFC ↓	[14]
CUMS C57BL/6 mice	EA	ST36	2 Hz and 0.5 mA,30 min, 4 weeks	SPT: sucrose preference↑, TST and FST: immobility time↓, OFT: total moved distance↑	Maximal number of intersections and length of astrocytic branches ↑, astrocyte-associated ezrin ↑	[15]
SNI C57BL/6 mice	EA	ST36, SP6	100 Hz, 0.3 mA, 30 min, 7 days	SPT: sucrose preference↑, TST and FST: immobility time↓, OFT: total moved distance↑	The BrdU^+^DCX^+^ cells / BrdU^+^ cells ratio ↑, the dendritic lengths of newborn GCs ↑, the expression of Tet1 and Prox1 in the ventral DG ↑	[16]
CUMS SD rats	MA	DU23, PC7	DU23 (3–5 mm), PC7 (2–3 mm), 3 weeks	body weight↑, SPT: sucrose preference↑, EPMT: open-arm time↑	The expressions of IL-6,CRP, and ACTH ↓; sum of intersections of dendrites ↑; the expressions of BDNF, PSD95, SYN, and PKMζ ↑	[17]
CUMS SD rats	MA	GV16, GV23	5 mm, 20 min, every other day for 28 days	body weight↑, SPT: sucrose preference↑, EPMT: open-arm time↑, FST: immobility time↓	The relative protein and mRNA expression of HMGB1 in amygdala ↓; the relative mRNA expression of TLR4 in amygdala↓; the % area of HMGB1 in amygdala↓; the relative expression of HMGB1, TLR4,IL-1β, and TNFα in spleen↓; the concentration of CRP, IL6, TNFα, IL-1β, CRH, ACTH and CORT in amygdala ↓	[18]
CUMS SD rats	MA	GV16, GV23	5 mm, 20 min, every other day for 28 days	body weight↑, SPT: sucrose preference↑, EPMT: open-arm time↑, FST: immobility time↓	The concentration of NO, cGMP, NF-κB p65,5-HT in the serum ↓, the concentration of 5-HT in the serum ↑; the expression of iNOS and nNOS in the LHb ↓, the expression of eNOS in the LHb ↑; the relative protein and mRNA expression of she and sIL2R in the LHb; the relative protein and mRNA expression of IL-4 in the LHb and liver ↑; α-diversity comparison of the gut microbiota ↑	[19]
CUMS SD rats	EA	GV20, GV29	2 Hz, 1 mA, 30 min, 14 days	SPT: sucrose preference ↑, OFT: total moved distance ↑	The accumulative percentage of spEPSC amplitude in vmPFC ↑; the expression of p‐DAT ↓; the expression of TAAR1 and PKA ↑	[20]
LPS C57BL/6 J mice	EA	L14, LR3	15 Hz,0.3 mA, 45 min,2 h before LPS injection, and 5 and 23.5 h after LPS injection	SPT: sucrose preference ↑, TST and FST: immobility time ↓	The level of IDO1, IL-6, IL-1β, and TNFα in brain ↓; the level of KYN, QA, and Glu in brain ↓	[21]
CMS C57BL/6 J mice	EA	GV20, BL23, KI 3	2 Hz, and 0.6 mA,15 min, 3 weeks	SPT: sucrose preference ↑, FST: immobility time ↓,	Nuclear/cytoplasmic ratio of NF-κB protein levels ↓; p-NF-κB,p-IκBα,NLRP3 ↓; the expression of IL-6, IL-1β, IL-18 and TNF-α ↓	[22]
LPS C57 mice	EA	LR3, GV29, DU20, L14	1 mA, 2 Hz/50 Hz, 20 min, 2 weeks	SPT: sucrose preference ↑, FST: immobility time ↓, OFT: total moved distance ↑	The ECM deposition ↓, the collagen accumulation in hippocampus ↓, the expression of MMP1/MMP9 ↑	[23]
CRS SD rats	MA	GV20, GV29	20 min, 21 days	SPT: sucrose preference ↑	The expression of TNF-α ↓, HMGB1 and IBA-1 in hippocampus ↓	[24]
CUMS Wistar rats	EA	GV20, GV29	1 mA, 2 Hz/15 Hz, 30 min, 2 weeks	SPT: sucrose preference ↑, OFT: total moved distance ↑	PFC neurons ↑, the expression of DDC ↑	[25]
CUMS SD rats	EA	GV20, GV29	0.6 mA, 2 Hz, 30 min, 14 days	SPT: sucrose preference ↑, OFT: total moved distance ↑	The relative protein and mRNA expression of FGF2 ↑, GFAP ↑	[26]
CUMS SD rats	EA	PC 6, SP 6	1–3 mA, 2 Hz, 20 min, 21 days	SPT: sucrose preference ↑, OFT: total moved distance ↑	The expression of c-Jun in hypothalamus and c-Fos in pituitary ↓; the expression of serum c-Fos and AP-1↓	[27]
CUMS SD rats	MA	GV23, PC7	20 min, 21 days	SPT: sucrose preference ↑, OFT: total moved distance ↑	5-HT in serum and hippocampus ↑; β-CaMKII mRNA and protein in hippocampus ↓; NMDAR mRNA and protein in hippocampus ↑; BDNF proteins in hippocampus ↑; GFAP ↑; Relative abundance of Bacteroidetes at the phylum level ↓; Bacteroidetes/Firmicutes ↓	[28]
CRS C57BL/6 mice	MA	KI10, LR8, LU8, LR4	Turned at a rate of two spins per second for 30 s, and then immediately removed,7 days	OFT: total moved distance ↑, FST: immobility time ↓	AST and CORT↓; TG56:8 ↑; leptin ↓; Ob-R positive cells in the ARC and CA3 ↑; NPY positive cells in the ARC↓; LXRα, SCD-1, FASN, HMGCR, and PPARγ ↓; Relative mRNA expression of IL-1β, TNF-α, and COX-2 in spleen ↓; the plasma leptin and AST ↓, IL-1β and TNF-α in liver ↓	[29]
CUMS SD rats	EA	LI4, LR3	15 Hz, 30 min, 3 weeks	SPT: sucrose preference ↑, OFT: total moved distance ↑, NSFT: latency time ↓	CaMK II protein levels in hippocampal ↓, NR2B in hippocampal ↓, fEPSP ↑	[30]
CUMS SD rats	EA	GV20, GV29, LI4, LR3	2/100 Hz, 0.2 mA, 20 min, 2 weeks	SPT: sucrose preference ↑, OFT: total moved distance ↑, NSFT: latency time ↓	AChE in the PFC ↑, ACh in the PFC ↓; spine density in PFC layer V pyramidal neurons ↑,the levels of BDNF, GluR1, GluR2, PSD95, and synapsin I in the PFC↑	[31]
CORT Swiss mice	ACE	GV20, GV14	On days 8 and 15	OFT: total moved distance ↑, EPMT: open-arm time ↑, TST and FST: immobility time ↓	5-HT and NE in serum ↑,ACTH in serum ↓;PLS-DA score; two up-regulated metabolites ↓;21 down-regulated metabolites ↑; mTOR signaling pathway ↑	[32]
SI SD rats	AC	DU20, EX-HN3, BL 23, BL20, BL 18, BL15, Ren4	Once	FST: immobility time ↓	CORT ↓, testosterone and estradiol ↑; BDNF in hippocampal ↑	[33]
CUMS SD rats	MA	GV23, GV16	5-6 mm, 20 min, every other day for 4 weeks	SPT: sucrose preference ↑, OFT: total moved distance ↑, FST: immobility time ↓	ROS in hippocampal ↓, hippocampal pyramidal neuron ↑; Bax, Bcl-2 and caspase-3 in hippocampus ↓; Nrf2 and HO-1↑	[34]
CUMS SD rats	EA	GV20, GV29	2 Hz, 1 mA, 5 V, 20 min, 2 weeks	SPT: sucrose preference ↑, OFT: total moved distance ↑	Autolysosomes in hippocampus CA1 neurons ↓; Expression of LC3 in hippocampus CA1 ↓	[35]
HIS SD rats	EA	ST36	dense wave, 1 mA, 30 min, 9 days	body weight ↑, thermal pain threshold ↑	Expressions of adrenal CORT↓, hypothalamus and serum CRH ↑; serum CORT ↓; alanine and valine ↓; glucose ↑	[36]
CUMS SD rats	EA	DU20, EX-HN3	2 Hz, 1 mA, 20 min, 28 days	SPT: sucrose preference ↑, OFT: total moved distance ↑	BDNF, TrkB, and tPA in hippocampus ↑; BDNF in Raphe Nuclei ↑; tPA mRNA in Raphe Nuclei ↑; BDNF mRNA hippocampus ↑	[37]
CUMS SD rats	EA	GV20, GV29	2 Hz, 0.6 mA, 30 min, 14 days	SPT: sucrose preference ↑, OFT: total moved distance ↑	5-HT1A mRNA and protein in hippocampal ↑	[38]
CUMS SD rats	EA	GV20, GV24	2 Hz, 0.6 mA, 60 min, 21 days	SPT: sucrose preference ↑, OFT: total moved distance ↑	CaM1, CaMKII and CaMKIV in the hippocampus ↑	[39]
CUMS SD rats	MA	GV16, GV23	the depth of 5 mm, 20 min	OFT: time in center↑, SPT: sucrose preference ↑, open-arm time in EPMT ↑, FST: immobility time ↓	The level of NO, cGMP and NF-κB in serum ↓, the level of 5-HT in serum ↑, the eNOS-positive cells in the LHb ↑, the iNOS- and nNOS-positive cells in the LHb ↓, serum AST and ALT levels ↓, the expression of sEH, and sIL2R in LHb↓, the expression of IL-4 in LHb ↑, α-diversity of the gut microbiota ↑	[40]
CRS C57BL/6 J mice	EA	HT7, LR3	2 Hz, 1 mA, 15 min, 7 days	CRS + EA (HT7): OFT: distance in center ↑, SPT: sucrose preference ↑, TST: immobility time ↓; CRS + EA (LR3): OFT: distance in center ↑, SPT: sucrose preference ↑, TST: immobility time ↓	EA (LR3): the neuronal activity of NAc shell ↑	[41]
CUMS C57BL/6 mice	EA	KI10, LR8, LU8, LR4	14 days	FST: immobility time ↓, SPT: sucrose preference ↑, OFT: activity time ↑	The expression level of Bax, Bcl-2, cGAS, STING, TBK1, IRF3, and NLRP3 in hippocampus ↓, TNF-α and IL-1β, in hippocampus ↓, 5-HT and NE in hippocampus ↑	[42]
SDS C57BL/6 J mice	EA	GV20, GB-34	2/100 Hz, 1 mA, 30 min, 21 days	SIR ↑, struggling time in FST ↑, total distance in OFT ↑, open-arm time percentage in EPMT ↑	The level of CB2R and AEA in hippocampus ↑	[43]
CRS SD rats	MA	GV20, GV29	20 min, 5 mm, 21 days	SPT: sucrose preference ↑,	The expression of IBA-1 in hippocampus ↓, the expression of TLR4、MyD88 and TNF-α in hippocampus ↓, IL-1β in serum ↓, IL-10 in serum ↑	[44]
CUMS SD rats, FS C57BL/6 J mice	MA	GU20, GV29, LI4, LR3	20 min, 5 mm, once on the 3rd day after drug intervention	CUMS rats: immobility time in FST↓ FST mice: immobility time in FST↓	CUMS rats / FST mice: the expression of M1-AchR in PFC ↓, the expression of GluR1, GluR2, BDNF, mTOR, p-mTOR, synapsin I and PSD95 in PFC ↑, the density of neuron dendritic spine ↑	[45]
CUMS SD rats	MA, ACE	GV23, GV16	MA:20 min,5 mm, once every other day within 28 days ACE: including days 1, 8, 15 and 22	MA: OFT: time in center↑, SPT: sucrose preference ↑, open-arm time in EPMT ↑ ACE: OFT: time in center↑, SPT: sucrose preference ↑, open-arm time in EPMT ↑	MA: the expression of MDA, IL-1β and TNF-α in serum↓, the expression of GSH, and GSH-PX in serum ↑, the expression of Sirt1, Nrf2 and GPX4 in hippocampus↑, the expression of p-p65 in hippocampus↓, Iron ion in serum↓, ACE: the expression of IL-1β in serum↓, The expression of GSH-PX in serum ↑, the expression of GPX4 in hippocampus ↑, the expression of TLR4 in hippocampus ↓	[46]
CUMS SD rats	EA	GV23, GV16	2 Hz, 0.5 mA, 20 min, 28 days	EPMT: open-arm time ↑, immobility time in the FST↓, sucrose preference rate of SPT↑	The expression of IL-18 in hippocampal↓, the expression of the SOD in hippocampal↑, SIRT1 in hippocampal↑, the expression of IBA-1 in hippocampal↓, the expression of Vglut1 in hippocampal↑, myelin sheath thickness↑, the expression of EphA4 In hippocampal↓	[47]
CUMS SD rats	EA	ST36, ST25	2/100 Hz, 0.7 mA, 30 min, 14 days	sucrose preference rate of SPT↑, immobility time in FST↓, the crossing score in OFT↑	Intestine propulsion rate ↓, the relative abundances of Bacteroidetes, Proteobacteria, and Actinobacteria↑, the relative abundances of Firmicutes ↓, the expressions of VIP in plasma↓, the expression of CGRP in plasma ↑, the expression of ACTH in plasma ↓, the expression of SST in plasma and colon↓	[48]
CUMS C57BL/6 mice	EA	GV20	2/15 Hz, 1 mA, 30min, 7 days	center time in OFT↑, immobility time in TST↓, sucrose preference rate of SPT↑	The abundance of *Actinobacteria*↑, the abundance of *Rikenella, Dubosiella, Ileibacterium, Bifidobacterium, and Allobaculum*↑	[49]
CUMS Wistar rats	EA	GV20, LR3	2 Hz, 2 mA, 20 min, 21 days	sucrose preference rate of SPT↑, center time in OFT↑, immobility time in FST↓	The expression of Igf2 gene and Cdh1 gene in hypothalamus↑, The expression of Mmp9 gene in hypothalamus↓	[50]

*HT7* Shenmen, *LR3* Taichong, *GB34* Yanglingquan, *GV23/DU23* Shangxing, *GV16* Fengfu, *BL18* Ganshu, *ST36* Zusanli, *SP6* Sanyinjiao, *PC7* Daling, *LI4* Hegu, *BL23* Shenshu, *KI3* Taixi, PC6 Neiguan, *KI10* Yingu, *LR8* Ququan, *LU8* Jingqu, *LR4* Zhongfeng, *GV14* Dazhui, *BL20* Pishu, *BL15* Xinshu, *Ren4* Guanyuan, *GV24* Shenting, *SPT* sucrose preference test, *OFT* open field test, *FST* forced swimming test, *TST* tail suspension test, *NSFT* novelty-suppressed feeding test, *EPMT* elevated plus maze test, *SIT* social interaction, *GAD67*glutamate decarboxylase 67, *PrL/IL* Prelimbic Cortex/Infralimbic Cortex, *PNN* perineuronal net, *PSD-95* Postsynaptic Density Protein-95, *NAc* nucleus accumbens, *PFC* Prefrontal Cortex, *NT-3/4/5* Neurotrophin-3/4/5, *iNOS* Inducible Nitric Oxide Synthase, *DCX*^+^ Doublecortin, *FNDC5* fibronectin type III domain containing 5, *TrkB* Tropomyosin-related kinase B, *CREB* cAMP response element-binding protein, *PKMζ* Protein Kinase M ζ, *LHb* Lateral Habenula, *BrdU*^+^ 5-Bromo-2'-deoxyuridine, GCs granule cells, *DG* dentate gyrus, *Tet1* Ten-Eleven Translocation 1, *Prox1* Prospero Homeobox 1, *CRP* C-reaction protein, *SYN* Synapsins, *HMGB1* High Mobility Group Box 1, *TLR4* Toll-like receptor 4, *cGMP* Cyclic Guanosine Monophosphate, *NF-κB* nuclear factor kappa-light-chain-enhancer of activated B cells, *nNOS* neuronal nitric oxide synthase, *eNOS* endothelial nitric oxide synthase, *She* Sclerostin, *sIL2R* Soluble Interleukin-2 Receptor, *spEPSC* spontaneous Excitatory Post-Synaptic Currents, *vmPFC* ventromedial prefrontal cortex, *DA* Dopamine, *TAAR1* Trace Amine-Associated Receptor1, *PKA* Protein Kinase A, *IDO1* Indoleamine 23-dioxygenase 1, *KYN* Kynurenine, *QA* Quinolinic acid, Glu Glutamate, *IκBα* Inhibitor of κB α, *ECM* Extracellular Matrix, *MMP1/MMP9* Matrix Metalloproteinase 1/9, *IBA-1* Ionized calcium binding adapter molecule 1, *DDC* Dopa Decarboxylase, *FGF2* Fibroblast Growth Factor 2, *GFAP* Glial Fibrillary Acidic Protein, *AP-1* Activator Protein 1, *CaMKII* Calcium/Calmodulin-dependent Protein Kinase II, *AST* Aspartate Aminotransferase, *Ob-R* Obestatin Receptor, *ARC* Arcuate Nucleus, *NPY* Neuropeptide Y, *LXRα* Liver X Receptor alpha, *SCD-1* Stearoyl-CoA Desaturase-1, *FASN* Fatty Acid Synthase, *HMGCR* 3-Hydroxy-3-Methylglutaryl-CoA Reductase, *PPARγ* Peroxisome Proliferator-Activated Receptor gamma, *fEPSP* field excitatory post-synaptic potentials, *LC3* Microtubule-associated protein 1 light chain 3, *Bax* Bcl-2-associated X protein, *Bcl-2* B-cell lymphoma 2, *Nrf2* Nuclear factor erythroid 2-related factor 2, *HO-1*Heme Oxygenase-1, *tPA* tissue Plasminogen Activator *AChE* acetylcholinesterase, *CaMK II* Calcium/calmodulin-dependent protein kinase II *cGAS* cyclic GMP-AMP synthase

In these studies, the most used acupuncture method was EA (29), manual acupuncture (MA) was used in 11, acupoint catgut embedding (ACE/AC) was used in 2, and MA and ACE were used in 2 study. In terms of acupuncture point selection, GV29/EX-HN3 (Yintang) and GV20/DU20 (Baihui) are still the most selected combinations, while the stimulating method of acupuncture is significantly different according to the design of each study.

## Impact of acupuncture on depressive physiology and pathology

### Neurotransmitter regulation

**Fig. 2:**
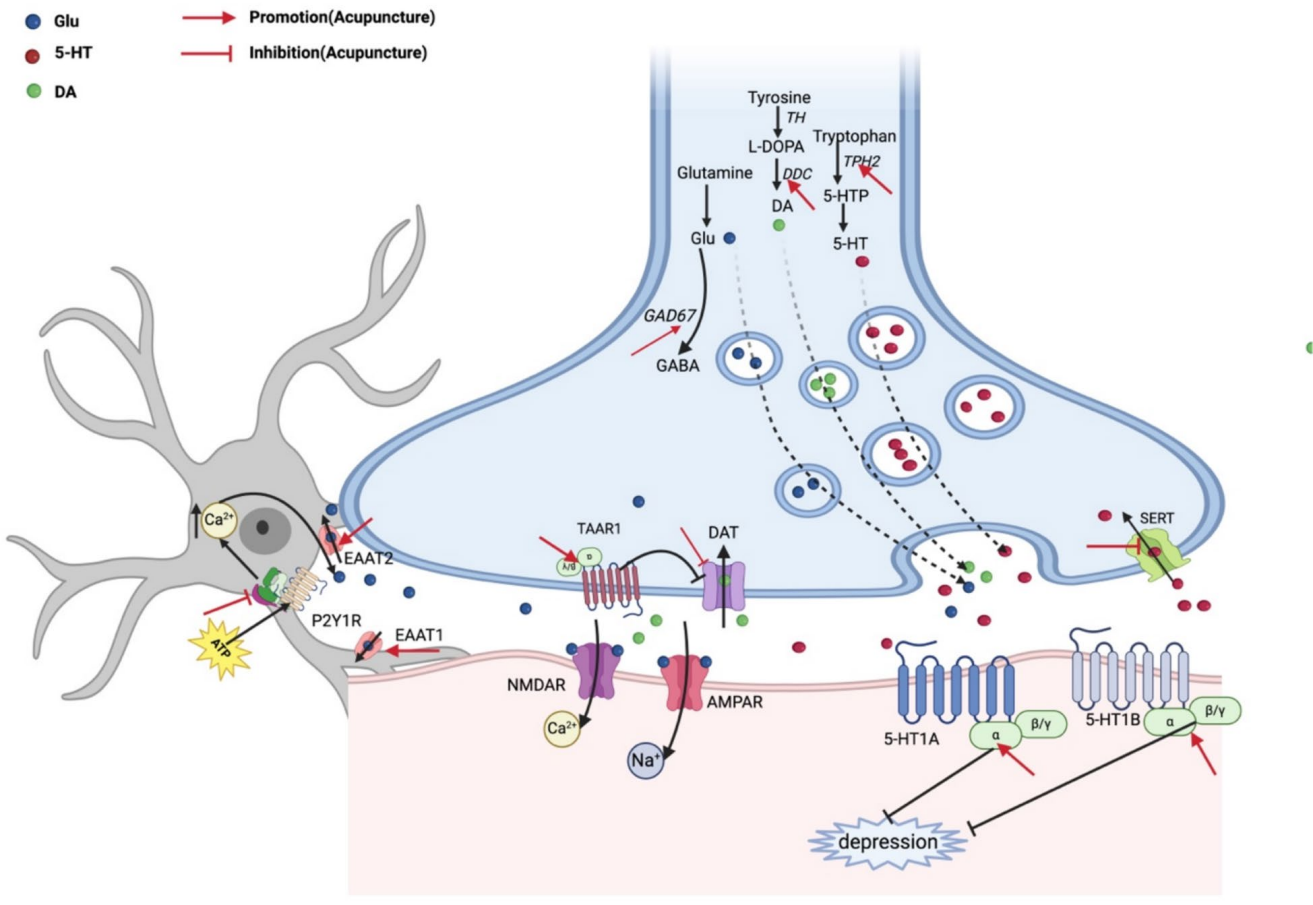
Acupuncture regulation of neurotransmitters

5-Hydroxytryptamine (5-HT) is the neurotransmitter most closely related to depression and is currently used as the target therapy for clinical antidepressant drugs. Serotonin transporter (SERT) is responsible for 5-HT reuptake by serotonergic neurons, and miRNA-16 is a post-transcriptional inhibitor of SERT. It has been reported that EA inhibits 5-HT reuptake through regulation of the mirNA-16-SERT pathway and can increase the concentration of 5-HT in the hippocampus and promote the formation of 5-HT by increasing tryptophan hydroxylase (TPH) [51, 52]. In addition, the 5-HT1A receptor and the 5-HT1B receptor are related to the release of 5-HT. Acupuncture treatment increases the expression of 5-HT1A in the cortex (Fig. 2), hippocampus, thalamus, and hypothalamus, as well as the expression of 5-HT1B in the cortex and thalamus, thus significantly alleviating depression-like behavior in mice [53, 54].

Glutamate (Glu) is an excitatory amino acid that is involved in the transmission of neurotransmitters. Glu levels may change in the brain in people with depression. Excitatory Amino Acid Transporter (EAAT) is mainly responsible for recycling excessive Glu from the synaptic gap to maintain the stability of neuronal activity. EA can increase the levels of EAAT 1 and EAAT 2 in the hippocampus of depressed rats, thereby alleviating depressive symptoms in rats [55, 56]. Glutamate decarboxylase 67 (GAD67) is a key enzyme in the conversion of Glu to the inhibitory neurotransmitter gamma-aminobutyric acid (GABA). EA can reverse the decreased GAD67 in the prelimbic cortex and infralimbic cortex (PrL/IL) of CUMS rats [7]. In addition, the P2Y1 receptor (P2Y1R) can regulate the release of a variety of neurotransmitters, including Glu. The level of P2Y1R in the hippocampus and prefrontal cortex (PFC) of SI-depressed mice is significantly increased, and EA can reduce the level of P2Y1R, thereby improving the resulting depressive behavior [9].

Dopamine plays an important role in emotion regulation, and increased activity of the dopamine transporter (DAT) can lead to excessive dopamine recycling. On the one hand, EA can improve depressive behavior in rats by up regulating the expression of the dopamine synthetase Dopa Decarboxylase (DDC) [25]. On the other hand, according to the study of Cai et al., EA can reverse the increase of total DAT and p-DAT in CUMS rats and alleviate depressive behavior, which may be through the activation of downstream proteins by Trace Amine-Associated Receptor 1 (TAAR1) to regulate the quantity or function of DAT [20]. Regarding other neurotransmitters, acupuncture has been shown to increase the expression of neuropeptide Y (NPY) in the hypothalamus [57]. EA can increase the level of Galanin (Gal) in the hippocampus of CUMS rats to a relatively normal level, increase the level of AChE in the PFC of rats, and reduce the level of Ach [31, 58]. All these effects can improve the depressive behavior of depressed model rats, but these studies have not been further studied and discussed.

In addition to the classic neurotransmitters related to depression mentioned above, recent studies have focused on the effect of acupuncture on the endogenous cannabinoid system (ECS). This study found that after 21 days of EA treatment, the content of CB2R (cannabinoid receptor 2) and AEA (anandamide) in the hippocampus of SDS mice increased significantly [43], but the specific pathway of EA affecting ECS is still unclear, and whether more CB1R in the CNS is affected by acupuncture also needs further exploration.

### Synaptic remodeling and neuroprotection

**Fig. 3:**
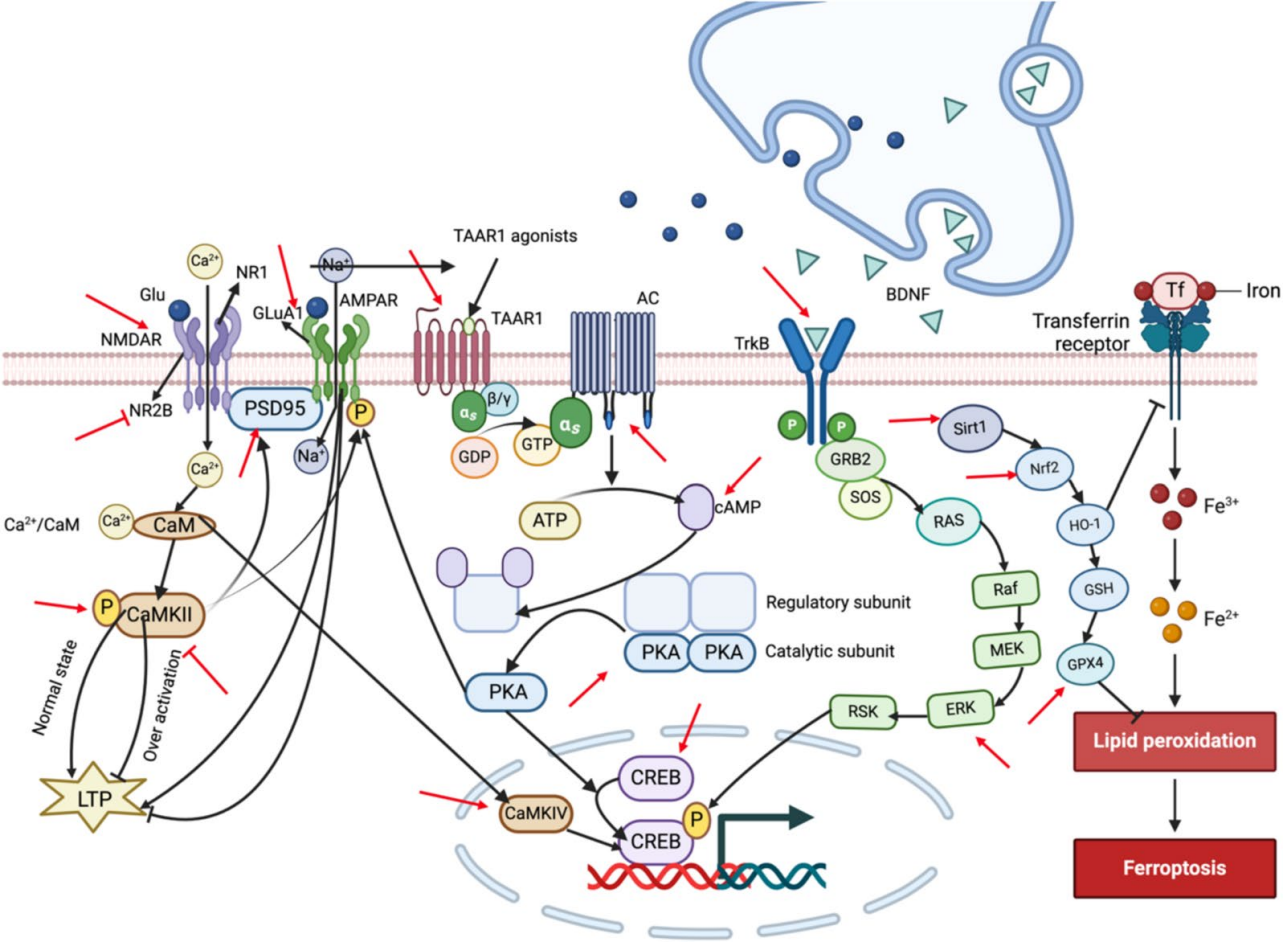
Synaptic remodeling and neuroprotective effects of acupuncture

Basic and clinical studies have shown that the reduction in the volume of brain regions involved in emotion regulation and cognition, including the PFC and hippocampus, and the reduction of neuronal synapses in these regions are closely related to depression [59]. Although current clinical drugs can block or reverse these neuronal defects to some extent, the efficacy of typical antidepressants is limited, and the response time is delayed by weeks to months. It is still necessary to further determine the neuronal and synaptic changes behind depression and explore the development of safer and more effective antidepressant therapy [60]. The most critical factor in the initiation of synaptic plasticity is the inflow of calcium (Ca^2+^) through NMDAR. The excessive or insufficient activity of NMDAR is related to the occurrence of depressive symptoms. CaMK II can be regarded as an indicator of intracellular Ca^2+^. When excessive Glu is combined with N-methyl-D-aspartate receptor subunit 2B (NR2B), a large amount of Ca^2+^ influx leads to excitatory toxicity and affects synaptic plasticity. Studies have shown that EA can down-regulate CaMK II and NR2B in the hippocampus and enhance Field Excitatory Postsynaptic Potentials (fEPSP) [30], while MA can up-regulate the expression of NMDAR in the hippocampus [28], which can significantly improve the depressive symptoms of rats (Fig. 3). GLuA1 is a subunit of the AMPA receptor and is involved in rapid synaptic transmission. PSD95 (postsynaptic density protein 95) is a protein located in the postsynaptic density region of neurons and is involved in the regulation of synaptic stability and plasticity. Perineuronal nets (PNN) are essential for neuronal stability, plasticity, and protection. The reduction of these three substances can be observed in the brain tissue of CUMS rats. EA can increase the protein expression of PNN cell density, GLuA1, and PSD95 in PrL/IL, regulate the expression of excitatory synaptic proteins, and reverse stress-induced GABA neuronal function damage to exert antidepressant effects [7]. EA can also up-regulate the levels of CaM1, CaMKII, and CaMKIV in the hippocampal calmodulin-dependent protein kinases (CaMKs) family, thereby regulating neural plasticity [39]. Mitochondria are important organelles in synaptic remodeling. CUMS can lead to the loss of post synapse density (PSD) in hippocampal neurons, mitochondrial enlargement, cristae dissolution, and disappearance, leaving empty membrane space. EA significantly changed 48 mitochondrial proteins and 16 synaptic proteins, which in turn promoted mitochondrial repair and reversed PSD loss caused by CUMS, thereby changing hippocampal synaptic plasticity [61]. In addition, cells can remove damaged organelles and proteins through autophagy, which plays an important role in neuronal health, and dysregulated autophagy can damage neuronal health and participate in the occurrence of depression [62]. Microtubule-associated protein light chain 3 (LC3) is a key protein in the process of autophagy. EA can reduce the increase of hippocampal LC3 caused by chronic stress and inhibit the level of autophagy to improve the depressive behavior of CUMS rats [35].

Brain-derived neurotrophic factor (BDNF) is a neurotrophic factor widely expressed in the brain. When BDNF binds to its receptor, TrkB, it can activate a variety of signaling molecules, including Ras. After Ras is activated, Raf is recruited to the cell membrane and activated by direct interaction with Ras. After Raf activation, MEK is further activated, and MEK re-activates ERK (extracellular signal-regulated kinase). After ERK activation, RSK (core body S6 kinase) can be further activated, and RSK directly or indirectly activates a variety of transcription factors, including CREB (cAMP response element binding protein). CREB is a key transcription factor involved in the regulation of a variety of gene expressions. These genes are involved in neuroprotection, memory formation, and neural plasticity. According to the results of several studies, MA can regulate a variety of neurotrophic factors, including BDNF and neurotrophic factor-3/4/5 (NT-3/4/5) [10], promote the BDNF/TrkB pathway in the lateral hypothalamus (LHb), up-regulate CREB, and maintain an appropriate ratio of pro-BDNF to BDNF [12]. EA enhanced hippocampal Raf, ERK, RSK, and CREB signaling pathway activity [63] and hippocampal p-ERK 1 and 2 [64]. On the other hand, the activation of TAAR1 can promote AC to catalyze the production of cAMP, activate PKA, and then activate CREB, while EA can increase the level of TAAR1 [20], enhance the activity of the AC-cAMP-PKA signaling pathway [65], and finally play the role of synaptic remodeling and neuroprotection under the action of CREB.

Collagen accumulation and extracellular matrix (ECM) deposition can interfere with the normal remodeling of neurons. MMP-1 and MMP-9 (matrix metalloproteinase-1 and 9) are involved in neural remodeling in the brain by decomposing ECM components. EA can up-regulate the levels of MMP1 and MMP9 in the hippocampus and reduce collagen accumulation and ECM deposition [23]. Li et al. also observed that the ratio of BrdU ^+^ DCX ^+^ cells to BrdU ^+^ cells (rate of new neurons generated) in the ventral DG region of the hippocampus in the EA group was increased, and the expression of Tet1 (ten-eleven translocation methylcytosine dioxygenase 1) and Prox1 (prospero homeobox 1) was increased [16]. In addition, Bax, Bcl-2, cytochrome C, cysteine-containing aspartate-specific proteases-3 (caspase-3), and apoptosis-inducing factor (AIF) are key proteins in the process of apoptosis. Excessive production of reactive oxygen species (ROS) can induce oxidative stress and cause cell damage. Studies have found that MA can reduce the expression of the above substances in the hippocampus and upregulate the levels of the antioxidant substances Nrf2 and HO-1 [34, 66]. Moreover, MA can also increase the levels of FNDC5 (a protein encoded by the fibroblast growth factor 21 gene) and its cleavage product, irisin, in serum. Irisin was found to be able to cross the blood–brain barrier and directly act on the brain, thereby promoting neuronal survival and nerve regeneration [12].

Ferroptosis is a new type of programmed cell death. It relies on iron ions and ROS to induce the accumulation of lipid peroxides (LPO). The mechanism involves the inactivation of glutathione peroxidase 4 (GPX4), which is the only enzyme used to reduce lipid peroxides in cells. The activity of GPX4 depends on reduced glutathione (GSH) [67]. Sirt1 is a NAD + -dependent deacetylase that activates Nrf2 (nuclear factor E2-related factor 2), which is a key transcription factor that regulates the cellular antioxidant response. On the one hand, Nrf2 can activate downstream GSH and GPX4.On the other hand, it can promote the expression of HO-1 (heme oxygenase-1). HO-1 is an enzyme with antioxidant and anti-inflammatory effects that ultimately inhibits the occurrence of ferroptosis [68]. Recent studies have found that acupuncture can increase the content of Sirt1, Nrf2, and GPX4 in the hippocampus of CUMS rats, reduce iron ions in serum, and prevent the occurrence of ferroptosis [46, 47].

### Inhibiting neuroinflammation

Stress events are the main factors inducing MDD, and long-term chronic stressors can inhibit immune function, resulting in an imbalance between pro-inflammatory and anti-inflammatory effects, which can induce inflammatory changes in the brain and peripheral immune system [69]. The related cytokines produced by inflammation can promote the occurrence of depression by affecting the production and release of neurotransmitters, the growth and survival of neurons, and the neuroendocrine and intestinal flora [70].

**Fig. 4:**
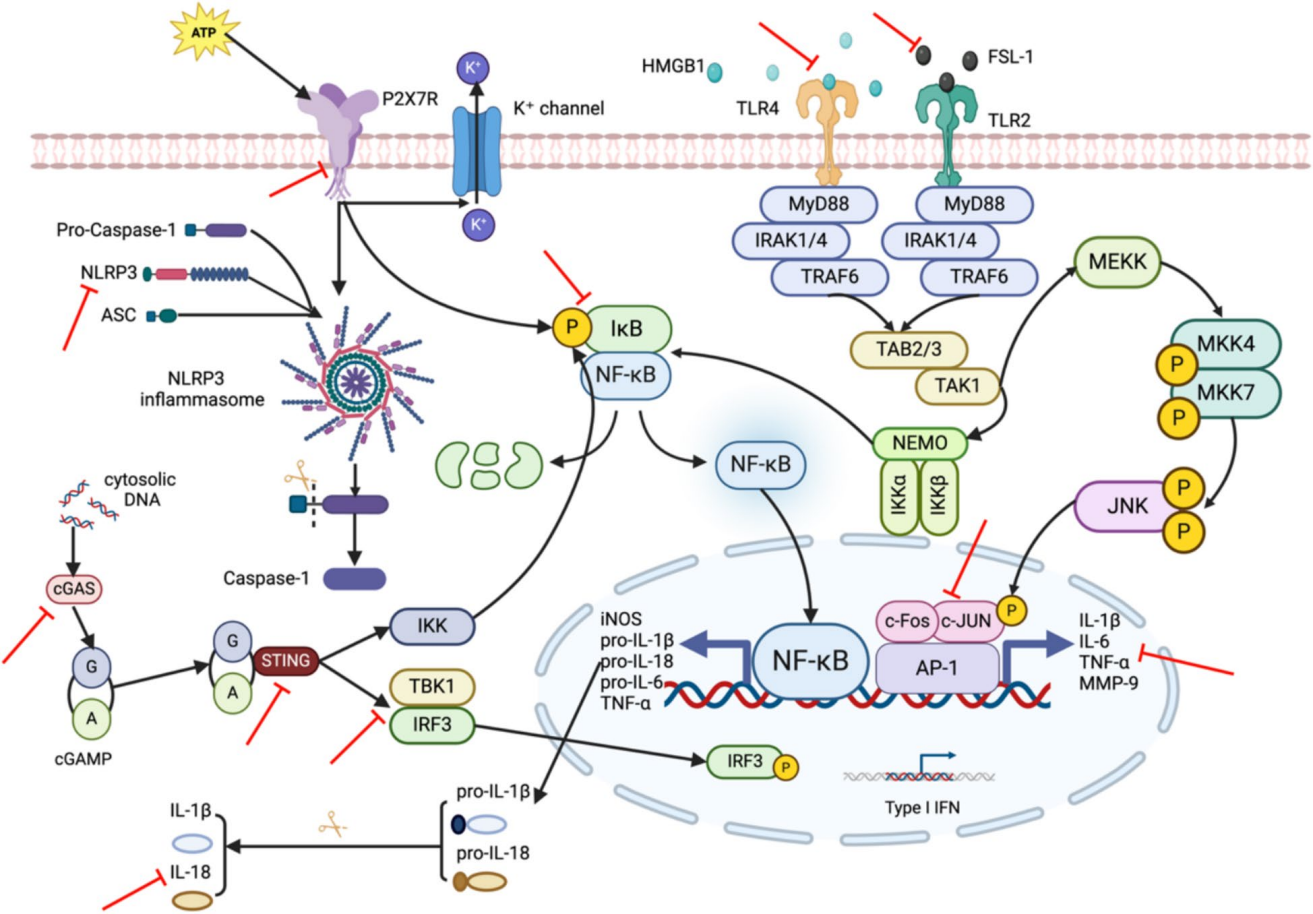
Inhibition of acupuncture on neuroinflammation

P2X7R is an ATP-sensitive ion channel located on the cell surface. Extracellular ATP acts as a ligand for P2X7R, and its activation can promote the assembly of the NLRP3 inflammasome, thereby activating caspase-1 and NF-κB signaling pathways. NF-κB can promote the expression of pro-inflammatory cytokines such as pro-IL-1β and inflammatory factors such as IL-6 and TNF-α. Caspase-1 can convert pro-IL-1β and pro-IL-18 into active forms of IL-1β and IL-18 [71]. Studies have found that EA can reverse the depressive symptoms caused by increased expression of P2X7R, NLRP3, IL-1β [14], IL-18, TNFα, and IL-6 [22] in brain tissue caused by chronic stress and reduce the expression of the microglial marker Iba-1 [72] (Fig. 4). Microglia are the main immune cells in the central nervous system (CNS) and are usually considered to be the main site of neuroinflammatory responses. Chronic stress can stimulate microglia to polarize to the M1 phenotype and induce depression by increasing pro-inflammatory cytokines [73]. Other studies have shown that EA can also reverse the overexpression of IL-6, IL-1β, and TNFα induced by LPS in brain tissue and serum and inhibit the inflammation-mediated kynurenine (KYN) pathway [21]. The main site of the KYN pathway is currently considered to be in microglia [74]. Astrocytes, another type of glial cell in the brain, are the main steady-state cells of the CNS. Chronic stress can lead to the morphological atrophy of astrocytes in multiple regions of the brain. Ezrin is a member of the ERM (ezrin, radixin, and moesin) protein family. It can help maintain and change the morphology of astrocytes by regulating the interaction between the cytoskeleton and cell membrane. Fibroblast Growth Factor 2 (FGF2) can be used as an effective mitogen to promote the proliferation and differentiation of astrocytes. Studies have shown that EA can not only increase the maximum intersection number and length of astrocyte branches by up-regulating the expression of Ezrin [15], but also up-regulate the expression of FGF2 (Fibroblast Growth Factor 2) to promote the generation of GFAP (astrocyte marker protein) [26]. In addition, another study has found that MA can up-regulate the level of GFAP in the hippocampus and forehead and increase the content of the serum anti-inflammatory cytokine IL-10 [75].

The NF-κB signaling pathway can also be triggered by a variety of other pathways. Under certain inflammatory conditions, the expression of inducible NOS (iNOS) increases, resulting in a significant increase in NO production. NO can promote the production of NF-κB and cGMP. As a second messenger, cGMP can participate in the occurrence of depression through a series of downstream effects. MA can reduce the expression of iNOS and nNOS in LHb and increase the level of eNOS, thereby inhibiting the production of NF-κB and cGMP and the depression caused by their combined reactions [19]. In addition, cGAS, as a DNA sensor in the cytoplasm, is activated when it detects double-stranded DNA (dsDNA) in the cytoplasm. The activated cGAS catalyzes GTP and ATP to produce cGAMP. cGAMP binds to STING (a stimulator of interferon genes) located on the endoplasmic reticulum membrane, undergoes conformational changes, and is transferred to the Golgi apparatus. STING interacts with IKK and activates IKK. The activation of IKK leads to the degradation of IκB, thereby releasing NF-κB and making it enter the nucleus. And the conformational change of STING can recruit and activate TBK1 (TANK-binding kinase 1) and then phosphorylate IRF3 (interferon regulatory factor 3). Phosphorylated IRF3 is transferred to the nucleus and initiates the expression of type I interferon and other inflammatory factors. EA can inhibit the inflammatory response caused by the activation of the cGAS-STING signaling pathway induced by CUMS, thus alleviating the anxiety and depression behaviors of mice [42].

Activation of Toll-like receptors (TLRs) can trigger a series of inflammatory reactions. Extracellular HMGB1 binds to TLR4, triggers TLR4 signaling, activates multiple downstream signaling pathways, including the NF-κB signaling pathway, and ultimately leads to inflammatory reactions. MA can inhibit the HMGB1/TLR4 pathway in brain tissue and reduce the concentration of inflammatory factors IL-6, TNFα, and IL-1β [18, 24]. Fibroblast-stimulating lipopeptide-1(FSL-1) induces an inflammatory response by activating TLR2, while EA can reduce the levels of FSL-1 and IL-1β in the rat hippocampus and increase the expression of the protective molecule transforming growth factor-β3 (TGF-β3) [76, 77]. The JNK (c-Jun N-terminal kinases) pathway is another inflammation-related signaling pathway induced by TLRs. c-Fos is a transcription factor that belongs to a member of the immediate early genes. It can bind to members of the Jun family, such as c-Jun, to form an activated protein-1 (AP-1) complex. The AP-1 complex can directly activate the gene expression of a variety of pro-inflammatory cytokines, such as TNF-α, IL-6, and IL-1. AP-1 can also regulate the expression of a variety of inflammation-related enzymes, such as cyclooxygenase-2 (COX-2) and matrix metalloproteinases (MMPs). EA can inhibit JNK signaling pathway-mediated neuroinflammation by down-regulating hypothalamic c-Jun, pituitary c-Fos, and serum AP-1 levels [27].

In addition, E-selectin, resistin, and G-protein-coupled receptor 55 (GPR55) are also considered to be associated with inflammation. E-selectin promotes the migration and activation of inflammatory cells by binding to specific ligands on the surface of leukocytes. Resistin can promote the release of pro-inflammatory cytokines (such as TNF-α and IL-6), while GPR55 is an atypical receptor in the ECS. The activated GPR55 may be related to the neuroprotective and anti-inflammatory effects regulated by ECS [78]. EA could not only down-regulate the expression of e-selectin and resistin in the rat hippocampus [79], but also reverse the decrease of GPR55 and the increase of inflammatory factors IL-6, IL-1β, iNOS, and TNFα caused by CSDS and promote the expression of anti-inflammatory factors IL-4 and IL-10 and the occurrence of hippocampal neurons [11].

### Regulate neuroendocrine and metabolic

The HPA axis (hypothalamus–pituitary–adrenal axis) plays a central role in neuroendocrine. Early stress can lead to continuous changes in the response of the HPA axis to stress in adulthood, resulting in increased susceptibility to depression [80]. The excessive activation of the HPA axis is one of the most consistent biological findings in depression. Patients with depression often show increased cortisol levels, increased pituitary and adrenal size, and increased activity [81]. Studies have shown that both EA and MA have the effects of regulating the HPA axis and acting as antidepressants. MA can reduce the levels of ACTH and CORT in the hippocampus serum of depressed rats and inhibit the disorder of the HPA axis induced by CORT in depressed rats [17, 57, 82]. EA decreased the expression of CRH mRNA in the hypothalamus and the levels of plasma ACTH and CORT [83]. The secretion of CORT is also closely related to circadian rhythm, showing obvious circadian rhythm changes. The disorder of circadian rhythm may be related to the poor mental status of patients with depression [84]. Maternal separation (MS)-depressed rats showed a decrease in circadian rhythm-related genes, but EA treatment could reverse the expression of these genes [85]. Similarly, EA treatment 1 h before CUMS stress modeling can reverse the body temperature and melatonin circadian rhythm disturbance caused by chronic stress and thus improve the depressive behavior in rats [86].

**Fig. 5:**
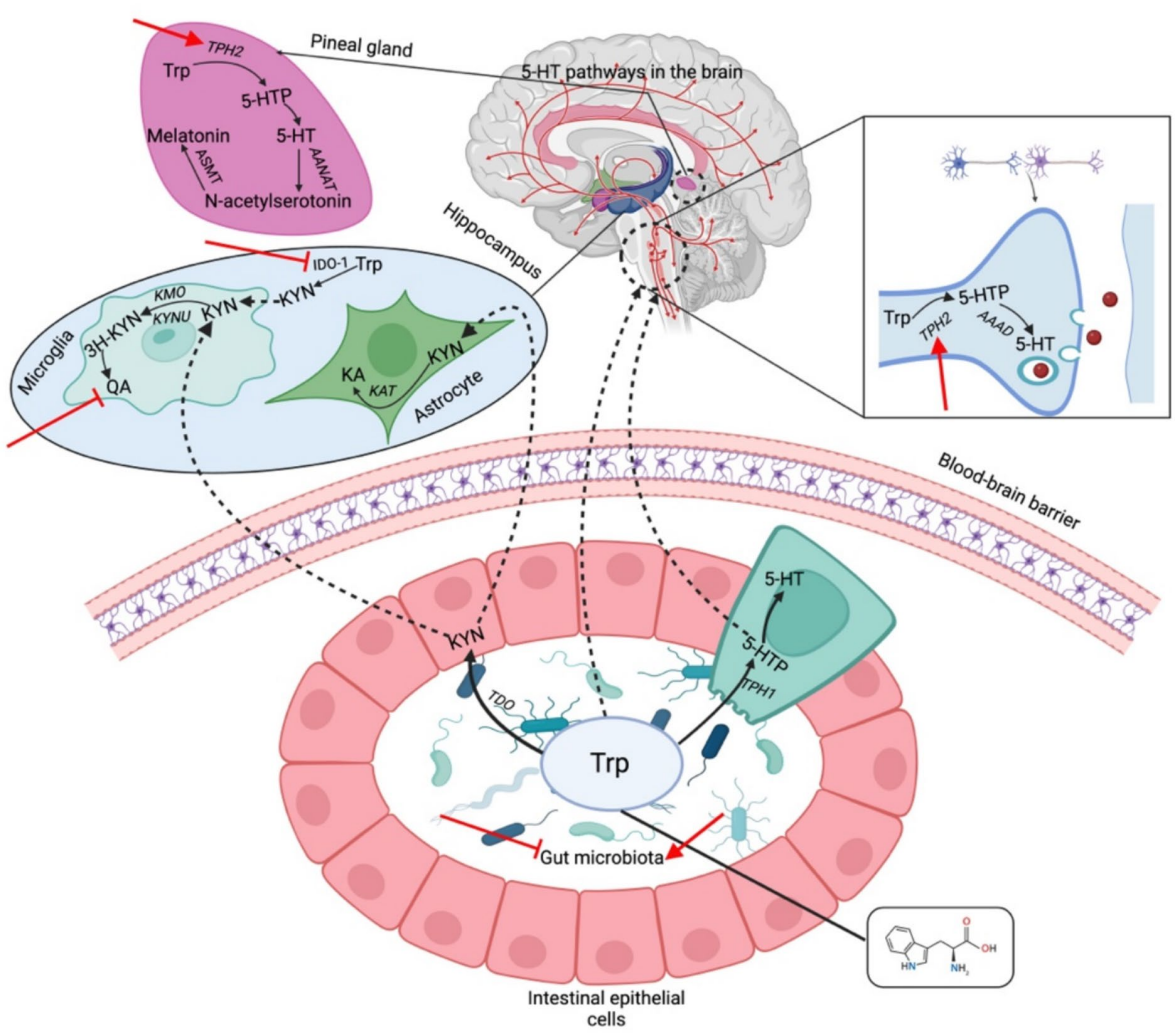
The regulation of acupuncture on neuroendocrine and metabolism

The metabolism of tryptophan (Trp) is also closely related to depression. About 1–2% of dietary Trp is absorbed through the intestinal tract and is catalyzed by TPH and aromatic acid decarboxylase (AADC) to generate 5-HT. About 95% of Trp enters the KYN pathway [87]. Trp is first catalyzed by IDO-1 to produce KYN in the brain. KYN can produce neuroprotective kynurenic acid (KA) under the action of kynurenine aminotransferase (KAT) and can also produce quinolinic acid (QA) under the action of kynurenine 3-monooxygenase (KMO) and kynureninase (KYNU). QA is an agonist of the NMDA receptor, which can mimic the effect of GLu and overactivate the NMDA receptor to cause nerve damage [88]. Studies have shown that EA can reduce the expression and activity of IDO-1 in brain tissue and reduce the levels of downstream products KYN, QA, and Glu [21] (Fig. 5). MA can also reduce the ratio of KYN/Trp in the hippocampus of mice and increase the serum KYN/3H-KYN ratio, thereby inhibiting the Trp- KYN pathway and significantly reducing depression-like behavior [89]. The normal intestinal flora is essential for maintaining intestinal function, which in turn deeply affects the metabolic process of Trp. Deng et al.'s research suggests that the disordered flora is significantly correlated with the behavior of CRS-depressed mice and the abnormalities of Trp metabolites [90]. Both EA and MA can regulate the intestinal flora disorder caused by CUMS. The former can increase the abundance of lactic acid bacteria and reduce the abundance of Staphylococcus [13]. The latter can reduce the abnormal relative abundance ratio of Bacteroidetes and Firmicutes caused by CUMS [29].

Considering the key role of Trp metabolism in depression, similarly, changes in other amino acid levels have also revealed their importance in mental health. Studies have shown that valine is positively correlated with anxiety, alanine is positively correlated with depression and suicidal ideation, and peripheral blood glucose is negatively correlated with depression [91]. EA can reverse the increase of alanine and valine levels and the decrease of serum glucose levels in depressed rats induced by HIS, thereby improving the chronic stress state of rats [37]. Higher copper and lower zinc levels were also observed in the serum of patients with MDD, which were correlated with the ratio of biochemical metabolites in the PFC and lentiform nucleus [92]. EA could reverse the significantly increased copper level and significantly decreased zinc level in the serum of rats induced by CUMS and alleviate depressive symptoms [93]. In addition, metabolomics analysis showed that ACE treatment regulated 23 differential metabolites in the brains of depressed mice and improved depression by reducing brain metabolic dysfunction [32].

## Discussion

In recent years, the basic and clinical studies of acupuncture treatment of depression, mainly represented by EA, have been increasing. The regulation of EA on the biological pathways of depression has been verified by the application of various animal models of depression, mainly focusing on the regulation of neurotransmitters, neuroplasticity and neuroprotection, the inflammatory response, and the neuroendocrine system. As one of the means of acupuncture, ACE and MA have also been proven to be effective for depression, but the frequency of use is far less than that of EA in both clinical and basic research. However, there is still a lack of research to confirm the differences in efficacy and mechanism of the three in depression. In addition, EA, as the most frequently used acupuncture method for depression, is mainly based on GV29/EX-HN3 (Yintang) and GV20/DU20 (Baihui), but there is also a lack of research to confirm the differences between different acupoints. On the other hand, through Table 1, we can see that there are significant differences in electroacupuncture parameters between different studies. Studies have shown that 2 Hz electroacupuncture has a better improvement effect on depression behavior caused by acute stress [94] while 100 Hz electroacupuncture has a better effect in dealing with pain-depression comorbidity [95], while the differences between other depression models and other frequencies remain to be confirmed.

As the main target of the current drug treatment of depression, the level of 5-HT can be significantly increased after EA intervention, and the level of 5-HT1A and 1B receptors in brain tissue can also be significantly improved after acupuncture [54]. As one of the main neurotransmitters in the brain, the 5-HT receptor system is huge and acts on different receptors to produce different effects. Receptors such as 5-HT1A, 5-HT2A, 5-HT2C, and 5-HT3 are associated with depression [96, 97]. Among them, 5-HT2A, 5-HT4, and 5-HT7 have been confirmed to participate in synaptic remodeling by affecting the ECM and MMP [98–100]. Therefore, based on the current research confirming the ability of EA to regulate the 5-HT system in depression, future research in this direction may focus on the effect of EA on 5-HT-related receptors and their effects. By promoting the formation and reconstruction of neurons and synapses, EA has a positive impact on the treatment of depression. Studies have shown that EA can increase the expression of GLuA1, PSD95 protein, and PNN cell density in the hippocampus and PFC, which are important factors for synaptic stability and plasticity. In addition, EA can also participate in the regulation of synaptic remodeling-related pathways such as BDNF, ECM, mitochondrial dysfunction, and oxidative stress. Heredity and genes lead to significant individual differences in depression. Studies have identified 17 independent gene loci that are significantly associated with depression, including genes related to postsynaptic, neuronal spine, and dendritic function [101]. Another study analyzed the transcription of Rho GTPase-related genes in the NAc. It was found that reducing the expression of Ras-related C3 botulinum toxin substrate 1 (Rac1) or inhibiting Rac1 activity in NAc increased social avoidance and anhedonia induced by social frustration [102]. In the past, EA lacked research on the genetic genes of depression. In the future, studies related to EA and synaptic remodeling in depression can be combined to explore related genes. Inflammation runs through a variety of mechanisms related to depression. There is a two-way interaction between the central inflammatory response and the HPA axis. The HPA axis can affect the expression of inflammatory factors and the activation of microglia. Microglia are also considered to be the main site of the Trp-Kyn pathway, and the intestinal flora determines the absorption of Trp and can affect the Trp-Kyn pathway in the CNS through peripheral Trp-Kyn metabolism [90]. Although the regulation of EA on depression-related inflammation, HPA axis, intestinal flora, and tryptophan metabolism has been confirmed, few studies have linked them together, and previous studies on the Trp-Kyn pathway have focused on the upstream of this pathway. The metabolic process of downstream Kyn-QA is still questionable [103, 104].

## Conclusion

In summary, according to the current basic research on acupuncture treatment of depression, acupuncture treatment represented by EA has multiple mechanisms to act as an antidepressant. However, future studies are needed to explore the differences between ACE, MA, and EA in the treatment of depression, as well as the operational parameters (such as frequency, intensity, duration, etc.) of EA in the treatment of different causes of depression. Although several treatment-related biomarkers and pathways have been identified, how these mechanisms interact and how they are linked to the pathophysiological processes of depression are still not fully understood. For example, recent studies have found that DA and 5-HT in the nucleus accumbens region regulate reward remodeling association learning in an adversarial way [105]. Future research can explore the role of this adversarial mechanism in the development of depression, as well as the influence of acupuncture on this adversarial mechanism.

## Data Availability

Every piece of data utilized in this systematic review is freely accessible to the public.
